# Development, Usability, and Efficacy of a Serious Game to Help Patients Learn About Pain Management After Surgery: An Evaluation Study

**DOI:** 10.2196/games.6894

**Published:** 2017-05-10

**Authors:** Brynja Ingadottir, Katrin Blondal, David Thue, Sigridur Zoega, Ingela Thylen, Tiny Jaarsma

**Affiliations:** ^1^ Landspitali - the National University Hospital of Iceland Surgical Services Reykjavik Iceland; ^2^ Division of Nursing Department of Social and Welfare Studies Linkoping University Linkoping Sweden; ^3^ Faculty of Nursing School of Health Sciences University of Iceland Reykjavik Iceland; ^4^ School of Computer Science Reykjavik University Reykjavik Iceland; ^5^ Department of Cardiology and Department of Medical and Health Sciences Linkoping University Linkoping Sweden

**Keywords:** evaluation studies, knowledge, pain management, patient education, self care, surgical procedures, operative, video games

## Abstract

**Background:**

Postoperative pain is a persistent problem after surgery and can delay recovery and develop into chronic pain. Better patient education has been proposed to improve pain management of patients. Serious games have not been previously developed to help patients to learn how to manage their postoperative pain.

**Objective:**

The aim of this study was to describe the development of a computer-based game for surgical patients to learn about postoperative pain management and to evaluate the usability, user experience, and efficacy of the game.

**Methods:**

A computer game was developed by an interdisciplinary team following a structured approach. The usability, user experience, and efficacy of the game were evaluated using self-reported questionnaires (AttrakDiff2, Postoperative Pain Management Game Survey, Patient Knowledge About Postoperative Pain Management questionnaire), semi-structured interviews, and direct observation in one session with 20 participants recruited from the general public via Facebook (mean age 48 [SD 14]; 11 women). Adjusted Barriers Questionnaire II and 3 questions on health literacy were used to collect background information.

**Results:**

Theories of self-care and adult learning, evidence for the educational needs of patients about pain management, and principles of gamification were used to develop the computer game. Ease of use and usefulness received a median score between 2.00 (IQR 1.00) and 5.00 (IQR 2.00) (possible scores 0-5; IQR, interquartile range), and ease of use was further confirmed by observation. Participants expressed satisfaction with this novel method of learning, despite some technological challenges. The attributes of the game, measured with AttrakDiff2, received a median score above 0 in all dimensions; highest for attraction (median 1.43, IQR 0.93) followed by pragmatic quality (median 1.31, IQR 1.04), hedonic quality interaction (median 1.00, IQR 1.04), and hedonic quality stimulation (median 0.57, IQR 0.68). Knowledge of pain medication and pain management strategies improved after playing the game (*P*=.001).

**Conclusions:**

A computer game can be an efficient method of learning about pain management; it has the potential to improve knowledge and is appreciated by users. To assess the game’s usability and efficacy in the context of preparation for surgery, an evaluation with a larger sample, including surgical patients and older people, is required.

## Introduction

Computer games as a medium for learning have been studied increasingly in recent years. Games have the potential to improve attention and motivation as players work on the challenges of the game [1]. “Serious games” is a term which refers to computer games that are designed with education in mind, either for learning or training [2]. Such games are used within health care to affect knowledge, attitudes, or behavior [3].

Serious games can facilitate adult learning with features such as interesting aims, goal-oriented problem-solving, active participation, and use of previous experience, and they can provide continuous feedback, which can stimulate motivation [4]. Furthermore, a debriefing on the performance of the player in the game can facilitate learning, by for example discussing the underlying reasons for choices that the player made in the game. These characteristics fit well with a current approach in health care that emphasizes the importance of patients’ empowerment and participation in their own care [5].

Within health care, serious games have been developed for educating both patients and health care professionals. For example, serious games have been tested with the goals of (1) improving patients’ self-care for diabetes, asthma, cancer, and Warfarin use and (2) improving diet, pain, mobility, lifestyle, and health-related knowledge [3,6-9]. Within this field, a recent study reported a successful validation of a framework that gamifies self-management of diabetes and its acceptance by patients [10].

Although still inconclusive, many studies on serious games within health care have reported positive outcomes. An example is the game Re-Mission that is intended to help young cancer patients improve their self-care. Players win by destroying cancer cells and other enemies in the body with weapons such as chemotherapy. The game was found to have significant effects on cancer knowledge [11]. Another game, SpaPlay, was designed to help women adopt healthier exercise and dietary behavior and evaluated in terms of effect on nutritional knowledge and body mass index (BMI). The evaluation study showed significant improvement in knowledge and decreased BMI [12].

Games have been used successfully as tools for managing pain, such as affecting the experience of pain and improving pain tolerance through distraction. Both commercial videogames [13] and games specially designed for pain management (eg, Snow World [14]) have proven to improve the pain experience for patients. Electroencephalography-based serious games have also been developed for use by patients, even at home, as tools to help manage their pain, offering a potential alternative to traditional drug treatment [15]. However, to help ensure that patients will use such tools to manage their pain in an effective manner, their knowledge of pain management needs to be improved. In particular, we view serious games as a convenient way to educate surgical patients about how to manage their postoperative pain.

Pain management is an area that currently needs improvement, since the prevalence of postoperative pain remains high, occurring in more than 80% of patients [16,17]. Pain is also common after hospital discharge, with 75% of patients reporting it, and of those, 80% rate their pain as moderate to severe [16,18]. Inadequate pain relief after surgery interferes with postoperative recovery, increases the risk for postoperative complications, increases the risk that the pain will become chronic, and has negative effects on quality of life [19].

Today, surgical patients are being discharged earlier than in previous years and same day–surgery accounts for nearly 70% of all surgery performed [20]. This has put increased responsibility on patients for self-care, including monitoring and treating symptoms such as pain. However, patients do not always follow the instructions they receive about pain management, and many avoid taking pain medications despite being in severe pain [21]. Patient-related barriers to effective pain management, such as their reluctance to report pain and use available analgesics, are well known, both within the population of patients with cancer [22] and patients undergoing surgery [23]. Improved patient education is vital to improve pain management and address such barriers, but providing patients with information alone is not sufficient [21,24,25].

The knowledge expectations of surgical patients are high [26,27] but are insufficiently met [27,28], and patients have requested improvements in this area [29]. They need to understand why managing pain is important and how they can be active participants in their own treatment [17,30]. Patients want information on how to treat their pain after being discharged, what to do if the treatment is insufficient, what side effects of medications to expect, and how to treat those side effects [31].

To pursue optimal health outcomes, there is a need to develop more effective educational interventions, and serious games have shown promising effects in the context of health care and patient education [8,11,12]. In a game environment, patients can not only acquire knowledge, but also move their trial-and-error learning from real life to the game’s virtual simulation. Also, the game provides a learning environment where attitudes, such as those that can hinder effective pain management can be explored, discussed, and potentially changed in collaboration with a health care professional.

During the early development and evaluation phase of a serious game, usability and efficacy are primary concerns. Usability is the extent to which a product can be used to achieve specified goals with effectiveness, efficiency, and satisfaction, and part of usability is the user experience that refers to the perceptions and responses to the anticipated use or after using the product [32]. User experience has both pragmatic and hedonic attributes [33]. “Efficacy” refers to the effect of an intervention on proposed outcomes; in the proposed study, we defined efficacy as the power or ability of the game to improve participants’ knowledge.

The aims of this study were, therefore, (1) to describe the development of a computer game for surgical patients about postoperative pain management and (2) to evaluate the usability, user experience, and efficacy of the game.

## Methods

This study has a pre- and posttest design and data were collected using multiple methods, including questionnaires, direct observation by a nonparticipant observer, and short semistructured interviews.

### Development of the Game

In planning the development and evaluation of the game as an intervention we used the first 3 principles of the Intervention Mapping protocol (proximal program objectives, theoretical methods, and practical strategies, and design program) [34], and we intend to use principles 4 and 5 (adoption and implementation) in future work to prepare interventions in the real-life situation of the hospital environment. We also used guidelines on how to develop more effective games and how to conduct research on them [35].

The game was developed and evaluated in an Icelandic setting. The process took place from January 2015 to January 2016. An interdisciplinary team of computer scientists, game and graphic designers, nurse researchers, and clinical nurse specialists (with expertise in the nursing care of surgical patients, patient education, and pain management) collaborated in the design and development of the game. The nurse researchers defined the clinical problem and its context and developed the idea of how a serious game could be used in patient education. The computer scientists and designers, who had expertise in game design and computer programming, contributed by transforming those ideas into a usable game to educate patients about pain management.

**Figure 1:**
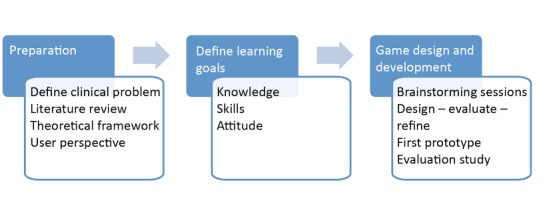
An overview of the development of the game.

The development of the game involved 3 phases: preparation, defining learning goals, and game design and development (Figure 1).

#### Phase I: Preparation

The preparation of the game design began by choosing and defining the clinical problem that the game was to address and searching the available literature for similar games.

The game is intended to help adult patients learn about pain management, a common self-care activity after surgery. Adult learning theories and the middle-range theory of chronic illness [36] were therefore chosen as the guiding theoretical frameworks. From Knowles’ theory on adult learning [37], we acknowledged the notions that adult learners are self-directed, they bring their previous experience to the learning, they need to see applications for new learning, and their active participation should be encouraged. From Kolb’s experiential learning theory [38] we incorporated the notion that learning occurs through concrete experience, observation and reflection, abstract conceptualization, and active experimentation. Finally, monitoring symptoms, such as pain, and managing them are the core concepts of self-care, and the importance of reflection and decision making is emphasized in the middle-range theory of chronic illness [36].

We integrated data from our previous qualitative study on patients’ experiences and their perceptions about both traditional and novel methods to learn about postoperative pain management, including serious games [39]. In that study, patients described unfamiliarity and skepticism toward the use of computer games for educational purposes but they were simultaneously curious, interested, and willing to test such a game if they were invited to do so. It is important to have recommendations and support from health care professionals in the use of such a novel method, and it must be simple to use as patients’ cognition may be impaired due to the surgical experience [39]. These findings were considered when designing the story of the game and the interface.

#### Phase II: Defining Learning Goals

The learning goals of the game were based on scientific literature regarding pain management and on the expertise of the nurses in the research team. The main learning goal was to improve knowledge about common pain medications that are frequently prescribed after surgery, including how they work, their dosages, effects, and side effects [31]. Additionally, other nonpharmacologic measures to treat pain were introduced, such as rest and distraction. Finally, a numeric rating scale (NRS; 0-10), frequently used in hospitals to teach patients to assess pain severity of patients, was included in the game. This reflected pain intensity and gave users feedback on pain management activities.

#### Phase III: Game Design and Development

The game was programmed in the C# programming language using the Unity game development environment [40] to be played on Android tablet computers. Table 1 presents the characteristics of the game.

**Table 1:** Characteristics of the game.

Characteristics	Description
Health topic	Self-care of surgical patients: pain management
Target players	Adults having surgery
Timing	Introduced as part of preparation before surgery and used again after surgery as part of discharge education
Game idea	A serious game intended to educate about facts concerning pain medication and strategies for effective pain management in the home environment after hospital discharge
Guiding theoretical framework	Middle-range theory of chronic illness, adjusted for surgical patients [36], adult learning [37], experiential learning [38]
Type of game	Realistic, educational, simulation
Intended outcomes	Knowledge about 4 commonly used pain medications after surgery (name, dosage, effects, possible side effects) Knowledge about nonpharmacological strategies for pain relief (rest, distraction) Knowledge about effective strategies for pain relief Problem-solving skills to control pain intensity Self-care pain monitoring skills Self-care pain management skills Facilitating attitude toward pain management
Levels of play	One game session consists of 3 games, each covers a 24-hour day (from 9 am to 9 am next day) with separate goals, and ends with an after-action review
User interface and platform	11.5″ touch screen on a tablet computer (Android) allows for easy use in the hospital environment Interface: Numeric rating scale for pain Pain medication board Button for showing goals Board for daily tasks	
Avatar	Human character who can walk around the house, use a shower and toilet, cook food, watch television, use a computer, rest on a sofa, lie in bed
Virtual environment (setting)	A house with a living room, bedroom, kitchen, bathroom
Software	Unity3d (Unity Technologies)
Estimated play time	30 minutes

Brainstorming sessions were used to ensure that the educational components of the game idea were accurately translated into the design of the game and to develop solutions for the interface, the continuous feedback system, and the after-action review. Such sessions were repeated, and the design of the game refined until a prototype was ready to be evaluated. To help pursue the identified learning goals, the adult learning principle that adults want to learn what is useful and relevant [38] was used to choose the game’s story, setting, and core interactions.

##### Story and Setting

The story of the game was designed to be a simulation of a relevant real-life situation, where the player’s character (avatar) has returned home from the hospital after having had surgery. By making different decisions about the character’s daily activities (eg, choosing between pain medications, performing basic household tasks, and taking time to rest), players can observe how their decisions influence the character’s recovery.

**Figure 2:**
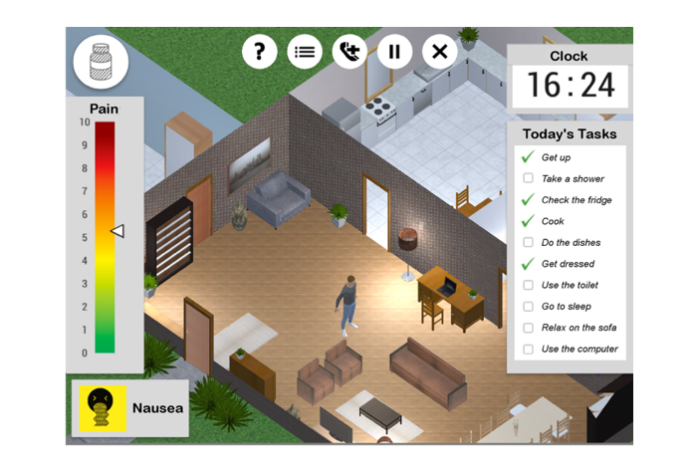
The game’s interface. Below center: the player’s avatar. At left, from top to bottom: button to access pain medication, current Pain Level indicator, current Side Effect. At right, from top to bottom: Current in-game time and listing of player tasks for the current day. At top middle, from left to right: buttons to review the tutorial, review the day’s primary objective, call the (fictional) hospital for help, pause the game, and exit the game. Screenshot translated from Icelandic.

The setting (game environment) was designed to look and react like a typical (Icelandic) home, to improve its familiarity to the game’s intended audience. Figure 2 shows a screenshot of the game environment with surrounding interface elements; the interface elements will be discussed later on.

##### Core Interactions

The game’s interactions were designed to simulate 2 types of activities that are highly relevant for recent surgery patients: (1) keeping up with the activities of everyday life, including household chores (eg, doing the dishes) and regular self-care (eg, taking a shower) and (2) managing their postoperative pain through various methods (eg, taking medication, resting, or enjoying distractions). To simplify the user interface, every activity was designed to be accessible with only a few taps on the screen (eg, tapping on the kitchen sink will cause the avatar to walk to the sink and do the dishes).

Experiential learning [38] emphasizes learning by doing. To apply this theory in the context of the game, we ensured that all of the game’s interactions are driven by an underlying, scientifically informed model of pain and the effects of different medications.

##### Pain and Medication Model

**Figure 3:**
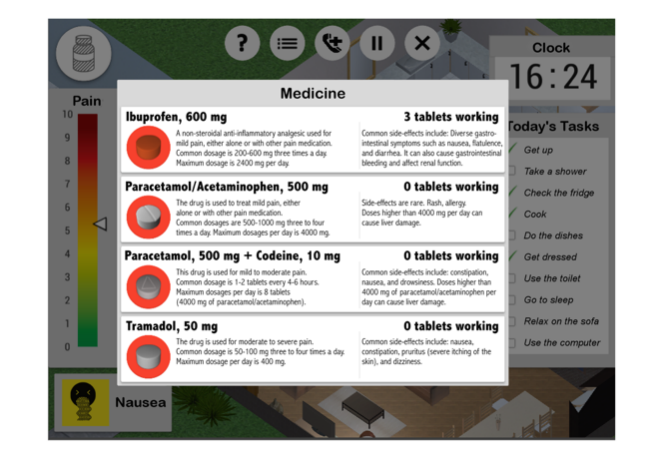
The medication board: by tapping the tablet icons (marked with red circles), the player can choose between 4 different pain medicines and read about their effects, possible side effects, and how many tablets are currently in effect. Screenshot translated from Icelandic.

A computational model of pain and medication effects was designed for the game using both scientific data and professional expertise. The model controls how each activity affects the avatar’s pain level, as shown by the NRS at the left of Figure 2. For example, any medication taken will decrease pain after an onset time, but it will lose effectiveness over time (Figure 3). Medication can also trigger the occurrence of side effects under conditions where they are known to be likely (eg, nausea can result from taking excessive amounts of codeine). Side effects appear both as icons in the interface (Figure 2) and as unique animations on the player’s avatar.

As the avatar’s pain becomes more severe, the model causes their mobility to decrease, making it more difficult to perform the activities that require movement around the house. Furthermore, the model occasionally and randomly simulates a real-life situation in which the pain becomes unmanageable (NRS ≥8) and does not respond to pain medication. The help button (middle button, top of Figure 2) gives contact with a (fictional) health care provider, and after the consultation the pain intensity decreases to 5 (representing the patient having received and implemented some helpful advice).

##### Objectives and Motivation

The primary value of the pain and medication model is that it allows patients to learn useful information through exploration and discovery without risking their immediate health; they can try out different courses of action in a safe, virtual environment, including those that might be harmful if they were performed in real life. Self-care theory [36] also holds that it is important for self-care learning to provide multiple opportunities to practice monitoring pain intensity and making pain management decisions. To promote players to practice and explore different alternative types of decisions, each game session consists of playing through 3 days in the avatar’s life, and on each day, the player is encouraged to pursue a new set of goals, some of them extreme. Specifically, 3 particular goal sets were chosen (one for each day) to encourage players to explore a wide range of different pain management strategies; they were

Day 1: “Take as little pain medication as possible”

Day 2: “Keep pain severity under 3 on an NRS regardless of side effects from pain medication”

Day 3: “Keep pain severity under 5 the whole day”

To motivate players to pursue the given goals, each player is given a rating from 1 to 3 stars at the end of each day, indicating how well they succeeded at achieving the goals of that day. Each day ends after a preset amount of in-game time has passed.

To motivate players to perform the daily tasks around the house and provide an additional avenue for feedback, the avatar occasionally produces small bubbles of text that represent the character’s (fictional) “inner monologue.” They appear both at random (for fun) and to provide information about the avatar’s pain status (eg, “I wonder what’s on TV?” or “I can feel the medication working…”).

##### Outcomes and Debriefing

**Figure 4:**
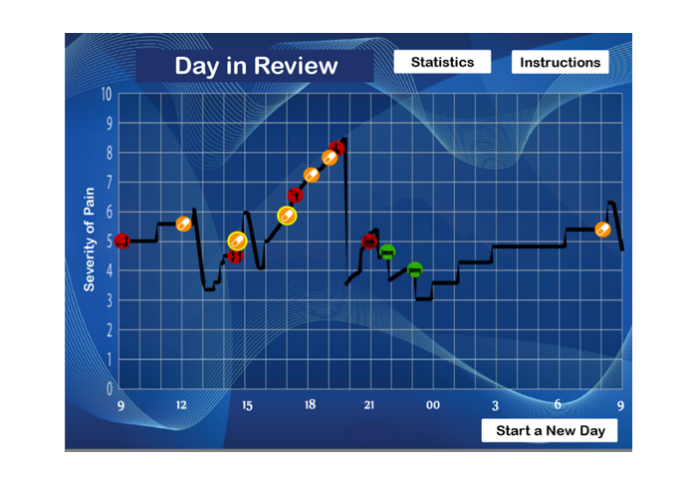
A panel showing a timeline of the previous in-game day. The black line shows the progression of the avatar’s pain level over time. Red nodes indicate activities that impaired pain relief, orange nodes indicate doses of medication, and green nodes indicate activities that relieved pain. Yellow outlines show the occurrence of side effects. Screenshot translated from Icelandic.

Learning through debriefings, where a learner is encouraged to review and analyze his or her experience after the fact, provides a fundamental link between the experience of playing and learning [41]. According to the literature, debriefings should focus on at least 3 elements: (1) what was done in the activity, (2) how well the activity worked for the learner, and (3) how the learning could be applied [42]. To support this kind of learning in the game, a mechanism was designed to record a log over time of 2 sets of information: the progression of the avatar’s pain level and the time and identity of each activity that the player performed (including both task completion and medication consumption). At the end of each in-game day, a graph of information appears (Figure 4) that overlays these 2 sources of information, allowing the player (potentially with assistance from a health care provider) to review and analyze the events that occurred during the day.

### Participants

Participants in the study were recruited from the public via a Facebook advertisement (n=11) and through a snowball method (n=9). Included were adults who use computers in daily life, but health care professionals, people with chronic pain, and people using pain medication regularly were excluded. Those were excluded because they had more knowledge and experience of pain management, including use of pain medication, than the target group of patients who are expecting to have surgery. We included people with and without prominent health problems (other than chronic pain) to reflect the targeted patient population. For ethical and practical reasons, patients were not included in this first evaluation of the game. The study was approved by the Bioethics Committee of Iceland (VSN-15-164) and conforms to the Helsinki Declaration [43]. All participants gave their informed consent by signing a form that explained the study.

### Data Collection

Data were collected from December 2015 to January 2016 by the researchers BI and KB, who are clinical nurse specialists and experienced in both qualitative and quantitative research methods. The testing and the pre-post testing data collection were done individually in one session, which took place in a hospital office and lasted approximately 90 minutes. Baseline data was collected first, and then the participant received a tablet computer and a simple, oral explanation of how to play the game. The playing session was video-recorded and directly observed by the researcher, who also took notes during the observation (nonparticipatory observation).

After playing, the participant filled out a questionnaire and was interviewed by the researcher. The semistructured and video-recorded interviews lasted from 8 to 15 minutes. They covered 2 main topics: Knowledge acquisition (“Please describe what this game was about.” “How did you make decisions in the game?” “Did you learn anything new and if so, what?”) And, usability (“What do you think about this method of learning about pain management?” “What was easy and not so easy while playing?” “How did you perceive the game character (the avatar)?” “How can the game be improved?”).

### Measures

#### Usability

Usability was assessed with 2 instruments: AttrakDiff2 and the Postoperative Pain Management Game Survey (POP-MGS). AttrakDiff2 is an instrument used to evaluate an interactive product [44]. It consists of 28 7-step items whose poles are opposite adjectives, and each set is ordered into a scale of intensity. The instrument has 4 subscales, each with 7 anchored items, which measure pragmatic quality (PQ), attractiveness (ATT), and hedonic quality (HQ), including identification (HQ-I) and stimulation (HQ-S) [45]. Possible scores are −3 to +3. A high HQ-I score implies a high perceived capability of communicating identity to others, or how users identify with the software in social context. A high HQ-S score implies a high degree of perceived novelty, stimulation, and challenge, which encourages development of the user’s skills and knowledge. A high PQ score primarily implies high usability, (ie, that it is task-related and reflects usefulness and ease of use. The ATT score summarizes the whole experience of the software [33]. The Icelandic version of the AttrakDiff2 has previously been validated [45]. The internal consistency of the scale in this study (Cronbach alpha) was .75.

POP-MGS is an adjusted version of a previously validated instrument based on variables identified in the technology acceptance model [46]. The 12 items measure perceived ease of use and usefulness of a simulation software and the design of its interface. Response options range from 0 to 5 on a Likert-type scale, where 0 means “strongly disagree” and 5 means “strongly agree” (reflecting higher usefulness and ease of use). The internal consistency of the scale in this study (Cronbach alpha) was .83.

#### Efficacy

Efficacy was measured using the Patient Knowledge About Postoperative Pain Management questionnaire (PAK-PPM), a 15-item instrument specially designed for the purpose of this study and reflecting the educational content of the game. The multiple-choice questions are based on a literature review and the clinical expertise of the authors. The scale has 5 components of postoperative pain management: pain after surgery (2 items), common pain medications and their dosages (7 items), pain management strategies (2 items), side effects of the treatment (2 items), and what to do if problems arise (2 items). Each item offers 6 alternatives to choose from; one of them is the correct answer, and the remaining 5 (including one which is “do not know” to the item’s question) are incorrect answers. Scores are graded according to the proportion of correct answers. The face validity of the PAK-PPM was established in advance by administering it to 5 individuals, not included in this study, resulting in minor adjustment and the addition of one response option (“do not know”). The internal consistency of the scale in this study (the Kuder-Richardson formula 20) was .68.

#### Demographic and Background Data

Demographic and background data were collected with questions on age, sex, education, employment, chronic illnesses, the use of computer, smartphone and computer games in daily life, health literacy and attitude toward pain management. Health literacy was assessed with 3 screening items [47]. Attitude toward pain management was measured with Icelandic Barriers Questionnaire II, [48] a 27-item instrument which is divided into four subscales. The instrument was adjusted such that referrals to “cancer pain” were changed to “surgical pain.” Participants rate the extent to which they agree with each item on a 6-point Likert-type scale ranging from 0 (do not agree at all) to 5 (agree very much). Higher scores reflect higher barriers to pain management. Internal consistency (Cronbach alpha) in this adjusted version of the instrument was .91.

The whole battery of questionnaires was pilot-tested by 5 individuals, to verify that the adjusted instruments were easily understood. No changes were required after that.

### Data Analysis

#### Quantitative Data

Descriptive statistics (median and interquartile range [IQR]), frequencies, and proportions (%) were used to describe the sample characteristics, as well as knowledge, barriers to pain management, and usability as appropriate for nonnormally distributed data. The Wilcoxon signed-rank test was used to compare knowledge scores for the PAK-PPM total scale before and after testing the game. IBM SPSS-23 statistics were used for this analysis (IBM Corp). The video recordings of the playing session were analyzed by measuring how long time it took the participants to play the game, counting how often they needed assistance and how often they ran into problems while playing.

#### Qualitative Data

Qualitative data were collected from the observations (free text notes) and from the interviews (open-ended questions). Data were analyzed with a directed approach to content analysis to validate the results of the survey on usability and efficacy (knowledge acquisition). With this approach, codes are derived from theory or relevant research findings and defined before or during data analysis, thus supporting or extending the existing theory [49]. The 2 concepts of usability and efficacy were chosen as categories for coding beforehand. The 2 researchers (BI and KB) took notes while watching and listening to the video recordings from the observations and interviews and categorized them as either “usability” or “knowledge.” The content was discussed between the researchers until agreement was reached and a summary of the interview with each participant was written.

## Results

### Characteristics of the Participants and Patient-Related Barriers

We recruited 20 people in the study. Their median age was 45 (range 24-67), they all used computers (n=20) in daily life, and most used smartphones (n=18) and played games (n=14) on the computer. Seven people had chronic illness, and health literacy was high (Table 2). The total score of the Barriers Questionnaire II was median 2.03 (IQR 0.77) on a scale of 0-5, and the highest barriers were found in the “harmful effects” subscale (median 2.71, IQR 1.71), followed by “physiological effects” (median 2.38, IQR 0.90), “communication” (median 0.58, IQR 1.27), and “fatalism” (median 0.00, IQR 0.33).

**Table 2:** Characteristics of participants and results from health literacy screening.

Background			N=20
Age			Median 45 years (range 24-67)
**Sex**			
	Women		11
	Men		9
**Education**			
	Basic education (≤9 years)		2
	College		4
	University		14
**Employment**			
	Office/marketing		4
	Technology/development/research		4
	Education		3
	Management		2
	Servicing/catering/travel/industry		4
	Other		3
Chronic disease? (yes)			7
**Use of information technology in daily life (yes)**			
	Computer		20
	Smartphone		18
	Tablet		15
Play games in a computer, smartphone, or on a tablet? (yes)			14
**Health literacy screening^a^**			
	**How often do you have problems learning about your medical condition because of difficulty understanding written information? (n=19)**		
		Never	11
		Occasionally	6
		Sometimes	1
		Often	1
		Always	0
	**How often do you receive help with reading hospital material? (n=19)**		
		Never	12
		Occasionally	3
		Sometimes	2
		Often	2
		Always	0
	**How confident are you filling out medical forms? (n=20)**		
		Extremely	13
		Quite a bit	4
		Somewhat	1
		A little bit	1
		Not at all	1

^a^Brief questions to identify patients with inadequate health literacy [47].

### Usability and User Experience

The game session (including the introduction, the 3 games, and the after-action review) took 34 minutes on average. The attributes of the game measured with AttrakDiff2 received median scores above 0 in all dimensions; highest for attraction (median 1.43, IQR 0.93) followed by pragmatic quality (median 1.31, IQR 1.04), hedonic quality interaction (median 1.00, IQR 1.04), and hedonic quality stimulation (median 0.57, IQR 0.68). The items on ease of use and usefulness as measured with the POP-MGS received a median score of 3.00 (IQR 1.75) to 5.00 (IQR 2.00) (possible scores 0-5) for all items except “I did not have any technical problems using the game” (Table 3). The observation through video-recording during testing showed that 15 participants asked for help, each 1-5 times (median 2 times), usually because of technical problems such as the avatar freezing or getting stuck in walls or not being able to proceed from one in-game day to another.

In the interviews, participants confirmed the ease of use, and while some found it easy enough to give to older people: “My mother is 83 but I think she could use this” (male, 42 years), others found it unsuitable for the very old. All participants managed to finish the session with minimum assistance but the observation revealed that the people with good computer skills were quicker to grasp what to do and how. The observations noted participants’ engagement while playing, and both the survey and interviews confirmed that they enjoyed playing the game.

The perceptions of participants about the game character (avatar) differed. Most male participants could identify with the “young male avatar.” Some of the female players however perceived the avatar as a young male they were taking care of while others identified with it and perceived it as an “it” and without a specific gender.

**Table 3:** Ease of use and usefulness of the game (Postoperative Pain Management Game Survey (POP-MGS); score 0-5; N=20).

Items		Median (IQR^a^)	% of participants who fully agree
**Part 1: Ease of use**			
	I found it easy to learn to get the game to stop or start	5.00 (2.00)	55
	It was fun using this simulation	4.00 (2.00)	30
	The way in which information was presented on the screen was clear	4.00 (1.00)	15
	It was easy to learn how to use the game	4.00 (1.75)	20
	I found the activity easy to follow	4.00 (1.00)	5
	The quality of video was good	3.00 (2.50)	25
	I found the game easy to navigate	3.00 (1.75)	10
	I did not have any technical problems using the game	2.00 (1.00)	15
**Part 2: Usefulness**			
	If I had recently had surgery or was preparing for one and the postoperative period, it would be helpful to get feedback from an expert on my pain management	5.00 (0.00)	85
	I think the game would be a useful addition to other education about pain management	5.00 (1.00)	55
	I think a simulation like this might encourage people who are recovering from surgery to learn about pain management	5.00 (1.00)	60
	If I was recovering from surgery, I think the game would encourage me to learn about pain management	4.00 (1.00)	35

^a^IQR: interquartile range.

The participants suggested additions and improvements to the game, both on the game mechanics, such as receiving more continuous feedback, but also on other pain management strategies, which will be considered in the next version of the game. For example, they suggested adding more nonpharmacologic methods to relieve pain and methods to prevent and treat the side effects of pain medication.

### Knowledge About Pain Management

**Figure 5:**
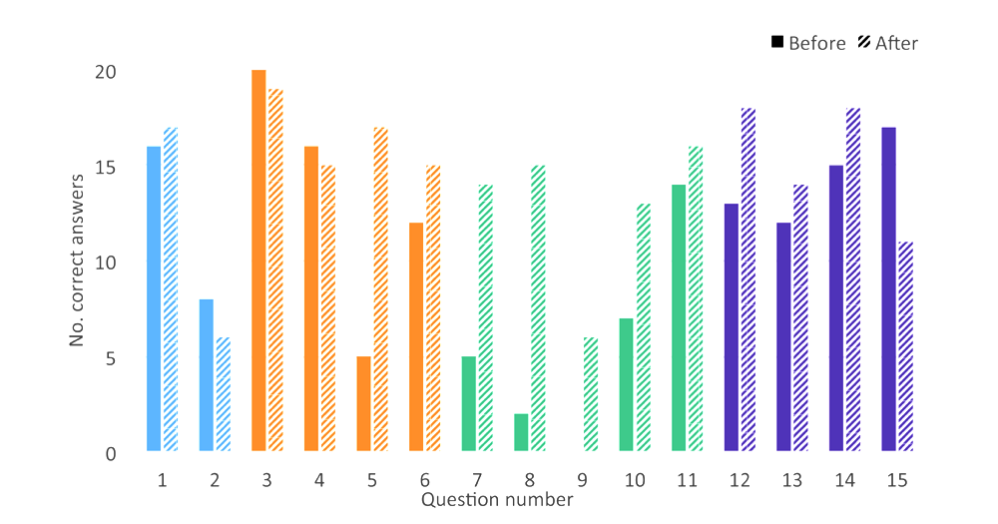
The number of correct answers for each of the 15 items in the knowledge test (Patient Knowledge About Postoperative Pain Management questionnaire) before and after playing the game. Questions 1-2 (blue) are about pain after surgery, questions 3-6 (orange) about different types of pain medication, questions 7-11 (green) about the dosages of the same medication and pain management strategies, questions 12-15 (purple) about medication side effects and how to react to unexpected situations.

From the questionnaire (PAK-PPM) we found that knowledge increased immediately after playing the game, from 54% correct answers before playing the game to 71% after (Z=−3.244, *P*=.001). Of the 20 participants, 18 improved their scores, one decreased his score, and one kept the same score. In 11 out of 15 questions the number of correct answers increased after playing the game (Figure 5). The number of correct answers increased most in items about pain medications and dosages. Smaller increases in the number of correct answers were found in items about postoperative pain and management strategies. In the item about seeking help in case of signs of complications (question 15), the number of correct answers decreased.

In the interviews, 16 participants said they had learned something new, while 4 said that they did not learn anything new because they had already acquired the knowledge from prior surgery and/or use of pain medication. Nearly half of the participants (n=9) said that one of the most important lessons learned was the importance of taking medications regularly instead of waiting for the pain to become intolerable, as they sometimes did in reality: “I learned the attitude that it is okay to take medication regularly, not to wait until the pain has reached the limit one can tolerate” (male, 40 years). They also learned about the effects and side effects of different medications and how to use multimodal pharmacologic and nonpharmacologic approaches (eg, that pain relief is promoted by resting or using distraction such as watching television or using the computer).

The participants also confirmed our theoretical assumption that they used previous knowledge from their own experience and life situations while learning with the help of the game: “I never chose the Ibuprofen because my doctor forbade me to use it when I had surgery” (female, 61 years).

## Discussion

This is, to our knowledge, the first paper describing the development and evaluation of a serious game that has the purpose of teaching surgical patients about postoperative pain management, including the use of pain medications and effective pain management strategies. The results of this evaluation indicate that a serious game may indeed be a useful and an attractive option for prospective surgical patients to learn about pain management.

### Efficacy

There was an increase in participants’ knowledge about pain management, especially about individual medication dosages and side effects, but also about pain management strategies such as regular medication intake and combining pharmacologic and nonpharmacologic approaches. Knowledge acquisition is the most common outcome measured in serious games in general [50] and studies have shown beneficial effects in such games within health care [51]. However, there has been a call for more advanced use of games focusing on affective, motivational and physiological outcomes, and behavior change outcomes in general to improve health-related outcomes [50,51]. The participants in this study were quite positive toward pain management and reported similar attitudes in the Barriers Questionnaire as both cancer patients and a sample from the public in previous Icelandic studies [48,52]. Their main concern seemed to be the harmful effects of pain medication in relation to surgery. In another study on pain experience and barriers to pain management, for Chinese patients undergoing thoracic surgery, even higher total barrier scores were reported, with the main concerns being pain medication tolerance, inhibition of wound healing, time intervals, and distraction [23]. The positive results from the current study indicate that our serious game can indeed be developed further to support more advanced health-related outcomes and address in more detail the misconceptions and attitudes that may hinder optimal pain management. For these purposes, the after-action review plays an important role and can be used as a starting point in the debriefing between patients and health care providers to initiate discussions about such barriers.

Although the game was able to improve the knowledge of the players, it remains uncertain if and how this type of learning can facilitate translation of knowledge into optimal behavior. Answering these questions would require a complex intervention study [53] with follow-up and well-defined long-term outcomes. Examples of outcome measures that would be important to measure are postoperative pain intensity, optimal recovery, knowledge, and satisfaction with patient education, with the participation of surgical patients. We are planning such a study as part of our future work.

### Usability

The participants in this study rated the usability of the game rather highly as confirmed both in the interviews and through the observations. They were engaged in the game the whole time and many enjoyed playing it. They also associated themselves with the avatar, either as themselves or as someone they sympathized with. It has been proposed that using avatars increases social presence, and allowing players to choose an avatar that they would like to represent them has resulted in greater satisfaction than when the only option is a standard male or female avatar [54,55]. Therefore, it should be considered that offering more choices of avatars could add to both engagement and the overall learning experience. Motivation is triggered by engagement and fun. The motivational appeal that games possess and which give them potential benefits in health education lie in their opportunities for active, exploratory, and experiential learning within a safe environment [8]. This is built into the design of the game: it is active because you need to complete the tasks, it is exploratory because of the different goals on different days, and it is experiential because of the consequences that are built into the mechanics.

The idea of playing a game to learn about pain management after surgery was well received by the participants in this study. They had different views on how appropriate it would be for older patients and those without computer skills, but nonetheless found it suitable in the case of prospective surgery (particularly for people with problems reading written material), and simple and easy enough to navigate for a wide range of users. Older adults are playing more games than in prior decades, for example, 26% of game players in the United States are 50+ years of age [56], and they report cognitive benefits and few difficulties in playing [57]. Age should therefore not be a hindrance for use with patients. However, the participants also confirmed findings from our earlier qualitative study [39] that although a game is an attractive addition to traditional methods, it should be introduced carefully and used under the supervision of health care providers. This supports our intention to introduce the game initially to patients before surgery (for practice purposes) and then again as part of their discharge education to support further learning.

### Strengths and Limitations

The participants in this study were recruited from the general public, but the results might be different if surgical patients tested the game. Several factors, such as anxiety and impaired cognition due to anesthesia and medications may affect their learning capabilities during the perioperative period [39]. However, the game is intended for patients undergoing surgery and, as theoretically all people may need surgery at some point in their lives, it was reasonable to assume that the participants in our study could sufficiently envision the situation of undergoing surgery and therefore act as real patients would. The participants had a wide variety of experience and skills in game playing and included people who design and evaluate games, as well as people with and without chronic diseases. While this group may not have been representative of surgical patients in general, it gave the evaluation an amount of variance that is feasible for such studies. On the other hand, it may also have caused some heterogeneity in the data, as the participants may have approached the game with different expectations. The sample size (n=20) was selected according to recommendations for collecting quantitative usability metrics [58]. Facebook was deemed a feasible channel to seek a wide variety of participants who fulfilled the inclusion criteria, as 74% of the Icelandic population are registered users of this social media network [59].

No validated instrument was available to measure changes in participants’ knowledge. The questionnaire designed for this study was useful in detecting changes in knowledge and had acceptable internal consistency, but it needs to be developed and validated further as it lacks established content and construct validity. Finally, since the postgaming test was implemented immediately after playing the game, we could not assess knowledge retention over a longer time.

### Conclusions

A serious game can be an efficient method to learn about pain management; it can improve knowledge and is appreciated by users. To assess the game’s usability and efficacy in the context of surgery, further development followed by an evaluation with a larger sample is required, including surgical patients, older people, and people with diverse health literacy.

## Abbreviations

ATT: attractiveness
BMI: body mass index
HQ: hedonic quality
NRS: numeric rating scale
PAK-PPM: Patient Knowledge About Postoperative Pain Management questionnaire
POP-MGS: Postoperative Pain Management Game Survey
PQ: pragmatic quality
